# The evolving role of creatine in public health: from food-based nutrient to supplement and beyond

**DOI:** 10.1017/S1368980025101390

**Published:** 2025-11-17

**Authors:** Sergej M. Ostojic

**Affiliations:** 1 Faculty of Health Sciences, University of Pecs, Pecs, Hungary; 2 Department of Nutrition and Public Health, University of Agder, Kristiansand, Norway

**Keywords:** Creatine, Functional foods, Dietary fortification, Nutrition policy, Public health

## Abstract

Creatine is a vital bioenergetic compound that remains largely overlooked within food systems despite its well-established role in human health and performance. Unlike creatinine – a downstream breakdown product of creatine metabolism commonly measured as a biomarker of kidney function – creatine functions as an energy buffer, facilitating the rapid regeneration of ATP in tissues with high metabolic demands such as skeletal muscle, brain and heart. Although a portion of daily creatine requirements is met through endogenous synthesis, dietary intake – primarily from animal-source foods – remains essential to maintain optimal physiological levels. Emerging evidence indicates that suboptimal creatine status, or creatine insufficiency, may be widespread, particularly among vegetarians, vegans, older adults, individuals with chronic illness and those with increased energy needs. This paper examines the evolving role of creatine across four domains: its natural occurrence in foods, incorporation into fortified food products, use as a dietary supplement and potential future classification as a pharmaceutical agent. Special emphasis is placed on differences in regulatory status, intended use, dosage, labelling and public health implications. In light of mounting evidence that creatine insufficiency may contribute to adverse outcomes – including impaired cognition, reduced muscular performance and vulnerability to stressors – integrating creatine into food policy and nutrition strategies represents a promising, scalable and preventive approach to improve population health.

Creatine is an amino acid-derived compound synthesised endogenously in the liver, kidneys and pancreas, playing a fundamental role in cellular bioenergetics^(1)^. By serving as a phosphate donor in the phosphocreatine system, creatine facilitates the rapid regeneration of ATP, particularly in tissues with high energy demands such as skeletal muscle, brain and heart^(2)^. It is important to distinguish creatine from creatinine, a breakdown product of creatine catabolism that is excreted in urine and commonly used as a biomarker of renal function; unlike creatine, creatinine does not contribute to energy metabolism. Although the human body produces a portion of its daily creatine needs, approximately 50 % must be obtained through the diet – predominantly from animal-based foods like red meat and fish^(3)^. While creatine is widely recognised for its use as a dietary supplement and ergogenic aid, its broader role within food systems remains underappreciated. In the current landscape of nutrition science, where functional foods, plant-based diets and personalised nutrition are gaining momentum, there is a pressing need to reconsider how creatine can be integrated into mainstream food policy and practice. This paper examines the evolving role of creatine across four domains: its natural occurrence in food, incorporation into fortified food products, use as a dietary supplement and potential future classification as a pharmaceutical agent. Emphasis is placed on differences in regulatory status, intended use, dosage, labelling and public health implications – particularly regarding strategies to address insufficient creatine intake through food-based approaches.

## Creatine requirements and population-level insufficiency

Creatine is considered a conditionally essential compound, with a daily requirement of approximately 1 g to maintain optimal physiological function (for a detailed review, see ref.no 4). Approximately half of this amount is synthesised endogenously, with the remainder expected from dietary intake. Animal-source foods are the primary contributors to exogenous creatine intake, making individuals who follow vegetarian, vegan or low-protein diets especially vulnerable to insufficient intake. Other at-risk populations include older adults, individuals with chronic disease, pregnant women and those with elevated energy demands, such as athletes.

Although severe creatine deficiency is rare and typically associated with inborn errors of metabolism^(5)^, a growing body of evidence suggests that nutritional creatine insufficiency – characterised by suboptimal but non-clinical levels – may be widespread^(6)^. Insufficiency can be assessed indirectly through circulating or urinary creatine concentrations, or more directly by measuring intramuscular creatine stores using magnetic resonance spectroscopy or biopsy-based assays^(7)^. In healthy young adults, plasma creatine concentrations typically range between 30 and 50 µmol/l, while intramuscular total creatine (free creatine plus phosphocreatine) averages 120–140 mmol/kg dry muscle^(7,8)^. Suboptimal levels are often defined as values that fall below these normative ranges, though no universally accepted clinical cut-off has been established. Vegetarians and vegans, for instance, often exhibit plasma creatine levels that are 20–30 % lower and intramuscular concentrations reduced by ∼10–20 % compared with omnivores^(9)^.

Population-level data suggest that creatine status tends to decline with advancing age^(7)^. Compared with younger individuals, older adults may have muscle creatine concentrations that are 15–20 % lower^(10)^, a reduction linked to impaired muscle strength, diminished cognitive performance and reduced resilience to stressors^(11)^. While some studies indicate that ageing may impair creatine synthesis and transporter function^(12)^, other age-related factors – such as decreased consumption of animal-source foods, reduced physical activity and the presence of chronic disease – likely contribute to modest reductions in creatine availability^(13)^. These combined behavioural and physiological influences may help explain why older adults often present with lower creatine stores and reduced responsiveness to supplementation, even though a consistent age-related decline has not been demonstrated across all studies. Importantly, such subclinical reductions are rarely detected in clinical practice, as creatine is not included in standard biochemical panels, and creatinine – the primary breakdown product of creatine – does not accurately reflect whole-body creatine status.

This emerging recognition of creatine insufficiency has important public health implications. It has been associated with higher risk of depression^(14)^, cognitive decline in the elderly^(15)^, cancer^(16)^, liver conditions^(17)^ and all-cause mortality^(18)^. Despite its biological significance, creatine is not currently included in nutrient intake recommendations, national dietary guidelines or food composition databases. Addressing this gap will require food system-oriented interventions, including targeted nutrition education, the development of creatine-fortified food products and the responsible use of dietary supplements. Such measures offer scalable and sustainable pathways to improve creatine status and support health across diverse population groups.

## Creatine as a food-derived nutrient

In its natural dietary form, creatine is found almost exclusively in animal-based products such as meat, poultry and fish^(19)^. In omnivorous diets, food-derived creatine contributes ∼1 g per d^(3)^, which, combined with endogenous synthesis, is generally sufficient to maintain tissue saturation in healthy individuals. Creatine’s function in this context is primarily to facilitate ATP regeneration through the creatine kinase system, enabling rapid energy buffering in high-demand tissues. Despite its importance, creatine is largely absent from current food system frameworks. Regulatory agencies do not require creatine content labelling, and it is rarely included in food composition databases or nutritional surveys. This oversight limits consumer awareness and hinders the development of public health policies aimed at optimising creatine intake through diet^(6)^. Moreover, while creatine from food is highly bioavailable, cooking and food processing can degrade its content, further reducing intake in some dietary contexts^(20)^. For individuals who consume limited animal products, this gap reinforces the need for food-based alternatives that can ensure adequate creatine status through accessible, culturally appropriate means.

## Creatine-fortified foods: a food system innovation

Creatine-fortified foods offer a novel strategy for addressing dietary insufficiency within the broader framework of functional nutrition. These products involve the targeted addition of synthetic creatine to plant-based or processed food matrices, such as meat alternatives, ready-to-drink beverages, protein bars and dairy substitutes. The aim is to increase creatine intake in populations that may not consume sufficient animal-source foods and to offer an accessible alternative to supplementation^(21)^. Fortification levels typically range from 0·5 to 3 g per serving, which is likely sufficient to support measurable increases in tissue creatine stores over time. From a regulatory perspective, these products often occupy a grey area. In regions such as the European Union, the addition of creatine to conventional foods may fall under Novel Food Regulation, requiring safety assessments and pre-market approval. While therapeutic claims are not permitted, general structure/function or nutrient content claims (e.g. ‘supports normal energy metabolism’) may be allowed. Importantly, creatine-fortified foods align with modern food system goals, including support for plant-based diets, preventive health strategies and personalised nutrition. They also provide an avenue for reducing reliance on supplements and increasing creatine intake in a format that is familiar, acceptable and integrated into everyday dietary practices. As such, creatine fortification represents a promising public health strategy that complements traditional dietary approaches.

## Creatine as a dietary supplement

Creatine’s most widely recognised application is as a dietary supplement, particularly in the form of creatine monohydrate. These products are extensively used to enhance muscular performance, cognitive function and support healthy ageing and are available in various formats including powders, capsules and functional drinks (for a detailed review, see ref.no 22). In many jurisdictions, including the USA, creatine supplements are regulated under food law (e.g. DSHEA) and do not require pre-market approval as long as they are not marketed with disease-related claims. Standard dosing involves an initial loading phase of 20 g per d for 5–7 d, followed by a maintenance dose of 3–5 g daily. This regimen has been consistently shown to increase muscle creatine content and improve performance in athletic, clinical and general populations. While creatine supplements are generally safe and well tolerated^(23)^, gastrointestinal discomfort may occur at high doses, and absorption efficiency diminishes with excessive intake. Despite their popularity, creatine supplements remain separate from core food systems and are often excluded from dietary policies and public health frameworks. In addition, public scepticism toward supplements – particularly among older adults and individuals seeking food-first approaches – can limit their reach. This highlights the need for complementary, food-based strategies, such as fortification, to promote broader adoption and ensure creatine sufficiency through accessible, regulated channels.

## Creatine as a pharmaceutical agent: a distinct pathway

Although creatine has not been approved as a pharmaceutical drug in any country, it has been investigated in clinical trials targeting a variety of health conditions, including amyotrophic lateral sclerosis, Parkinson’s disease, heart failure and depression. These trials, often conducted under Investigational New Drug (IND) frameworks or equivalent protocols, allow for scientific exploration of therapeutic efficacy without permitting commercial marketing as a drug. If creatine were to be developed as a pharmaceutical product, it would fall outside the domain of food systems entirely. Regulatory approval would require preclinical toxicology studies, Phases I–III clinical trials, GMP manufacturing standards and formal market authorisation via regulatory bodies such as the FDA or EMA^(24)^. Therapeutic formulations may involve higher doses than those used in supplementation – sometimes exceeding 50 g per d – and could be administered orally or intravenously. While the pharmaceutical pathway may be suitable for specific clinical populations, it introduces barriers such as increased cost, limited accessibility and dependency on healthcare systems. In contrast, food-based approaches – whether through natural intake, fortification, or supplementation – offer broader reach and align more effectively with public health objectives focused on prevention, equity and sustainability (Table 1).

**Table 1.** Creatine as food-derived nutrient, fortified food, supplement and pharmaceutical agent

Domain	Food-derived creatine	Creatine-fortified foods	Dietary supplement	Pharmaceutical agent
Source	Naturally present in animal-based foods (e.g. meat, fish)	Plant-based or processed foods with added creatine (e.g. bars, beverages)	Synthetic creatine in powders, tablets, capsules	Purified creatine formulated for therapeutic use (oral or IV)
Regulatory classification	Food ingredient	Novel or functional food (regulations vary by region)	Dietary supplement (e.g. DSHEA in the USA)	Drug (requires regulatory approval, e.g. IND/NDA)
Primary purpose	Supports normal dietary intake of creatine	Compensates for low intake, targets general or specific populations	Enhances physical/cognitive performance or fills dietary gaps	Treats or manages medical conditions (e.g. muscle wasting)
Claims allowed	No specific claims beyond nutrition labelling	Structure/function or nutrient content claims (e.g. ‘contains creatine’)	Structure/function claims only	Full therapeutic claims allowed (e.g. ‘treats sarcopenia’)
Dosage range	∼0·38−1·0 g/d	Typically 0·5−3 g per serving	Commonly 3−5 g/d (up to 20 g/d in loading)	May exceed 20−50 g/d in clinical settings
Bioavailability	Moderate to high (within food matrix)	High, depending on formulation and matrix	High at standard doses; saturable at high intakes (> 3−5 g)	Optimised for therapeutic effect; often IV administration
Population targeted	General omnivorous population	Vegetarians, elderly, athletes, general public	Athletes, ageing population, clinical users	Patients with specific diagnoses (e.g. ALS, congestive heart failure (CHF), sepsis)
Health system role	Maintains baseline physiological levels	Preventive or supportive nutrition	Enhances health and performance	Medical intervention
Safety monitoring	General food safety standards	May require novel food approval or monitoring in some jurisdictions	Post-market monitoring required	Requires clinical trials and pharmacovigilance
Examples of use	Meat, fish	Plant-based meat alternatives, creatine-fortified drinks/bars	Creatine monohydrate supplement powders	IV creatine for neuromuscular disorders (experimental use)

ALS, amyotrophic lateral sclerosis.

## Conclusion

Creatine occupies a unique and underutilised position within contemporary food systems. While traditionally associated with sports performance supplements, its biological importance and broad range of health benefits call for expanded integration into food policy and nutrition practice. Populations at risk of creatine insufficiency – including vegetarians, older adults and individuals with high energy demands – stand to benefit from targeted interventions that go beyond supplementation alone. Creatine-fortified foods, improved public guidance and the incorporation of creatine into national nutrition strategies represent viable pathways to improve health outcomes at scale. As interest grows in sustainable diets and food-first approaches to health, leveraging creatine as a functional nutrient within food systems offers a timely and impactful opportunity. Future research, regulation and innovation should focus on embedding creatine into the evolving landscape of food-based health solutions – bridging gaps in intake, reducing disparities and supporting health across the lifespan.
